# Hemophagocytic Lymphohystiocytosis in a Patient With Suspected Acute Myeloid Leukemia and Recent Viral Illness Compatible With Dengue: A Case Report and Literature Review

**DOI:** 10.1002/cnr2.70545

**Published:** 2026-04-14

**Authors:** Halima Saleh, Sariya Khan, Huda Albraim, Sahar Albraim, Amatul Rafey, Mable Pereira, Tarek Mohammed

**Affiliations:** ^1^ Department of Internal Medicine Saudi German Hospital Jeddah Saudi Arabia; ^2^ General Medicine Practice Program Batterjee Medical College Jeddah Saudi Arabia; ^3^ General Practice MyClinic Jeddah Saudi Arabia; ^4^ Deccan School of Pharmacy Hyderabad India; ^5^ School of Medicine Lincoln American University Guyana; ^6^ Department of Hematology and Oncology Al Andalusia Hospital Jeddah Saudi Arabia

**Keywords:** acute myeloid leukemia, dengue fever, hemophagocytic lymphohistiocytosis

## Abstract

**Background:**

Hemophagocytic lymphohistiocytosis, or HLH, is a very rare and aggressive disorder of the immune system, for which the trigger may be an infection, malignancy, or autoimmune disease. It has predominantly been described as triggered by T/NK lymphomas, but acute myeloid leukemia‐induced HLH is a very rare occurrence. Viral infections like dengue fever may act as potential triggering factors.

**Case Presentation:**

A 24‐year‐old male developed persistent high fever, weakness, and pancytopenia a few days after recovery following a recent febrile illness clinically compatible with dengue infection in a dengue‐endemic setting, supported by IgG seropositivity (IgM negative). He was found to be reactive for hyperferritinemia, coagulopathy, and hepatopathy. Bone marrow biopsies revealed blasts of 78%, which were suggestive of AML and myelomonocytic differentiation but also reflected abnormalities in the chromosomes *t*(1;9)(q42;q22), add(11)(q23).

**Discussion:**

This is an instance of an extremely rare and critical manifestation of HLH, coinciding with suggestive AML, seemingly triggered by an acute dengue illness. In our literature search, there were a few instances of AML and HLH, either preceding AML, concurrent, or post‐AML, mainly in younger individuals with high‐risk cytogenetics and an aggressive disease course. Generally, the prognosis in these instances turned out to be poor, mainly for those who experienced a delay in diagnosis and management or those complicated with infections and chemotherapy. In the given scenario, the patient suffered from severe complications in the initial stage, including disseminated intravascular coagulation, but responded to supportive care, and his clinical condition was stabilized. Later, the patient was shifted to a referral institute for management, and further prognosis information was not obtained.

**Conclusion:**

This particular case illustrates the importance of considering the possibility of HLH in the setting of unexplained systemic inflammation with the presence of an identified hematologic malignancy and preceding infection by a virus. However, the prognosis for patients with concomitant diagnoses of both HLH and AML is not encouraging.

## 1 Introduction

Hemophagocytic lymphohistiocytosis (HLH) is a rare but serious syndrome of inappropriate immune overactivation by hyperinflammation and breakdown of immune regulation. The syndrome results from uncontrolled growth and activation of cytotoxic T lymphocytes, natural killer (NK) cells, and macrophages, leading to massive tissue infiltration and cytokine storm, which culminate in multiorgan failure and dysfunction [1]. HLH may be inherited (familial HLH) or acquired (secondary HLH), the latter being brought about by infections, malignancies, autoimmune diseases, or drugs [2]. Although HLH is more familiar in the pediatric age group, its frequency in adults is increasing and poses challenging diagnostic and treatment tasks. Pediatric diagnostic criteria originally made by the Histiocyte Society for pediatric patients (HLH‐2004) have been widely used in adults, albeit with limited validation in adults [3, 4]. These consist of a subset of clinical manifestations and laboratory findings such as persistent fever, splenomegaly, cytopenias, hyperferritinemia, hypertriglyceridemia, hypofibrinogenemia, elevated soluble IL‐2 receptor (sCD25), and hemophagocytosis on tissue biopsy. In adults, however, these criteria may not always be fully met at presentation, and alternative tools such as the HScore are often used to support early diagnosis in clinically suspected cases [3, 5].

Of the secondary causes, malignancy‐associated HLH (M‐HLH) is very lethal and most frequently linked with hematologic malignancies, specifically T‐cell and NK‐cell lymphomas. In a large systematic review of 219 patients with M‐HLH, 93.7% were caused by hematologic malignancies, 35.2% by T/NK‐cell neoplasms, and secondarily by B‐cell lymphomas and other leukemias [6, 7]. Acute myeloid leukemia (AML), while less commonly implicated, has increasingly been found to be identified in case reports and small case series as an inciting or concomitant condition in the pathogenesis of HLH [8].

Dengue virus is another major infectious causative agent of HLH. Dengue‐associated HLH (D‐HLH) is a recognized complication, especially in endemic regions, and may occur in the febrile, critical, or convalescent stages of dengue infection. In such cases, HLH may be occult due to overlapping features such as fever, cytopenias, and transaminitis [9]. The synergistic action of a viral trigger in a patient with an inherent hematologic neoplasm can result in fulminant HLH and further complicate management and prognosis.

Here, we present an unusual and diagnostically challenging case of HLH secondary to infection associated with a recent viral illness compatible with dengue occurring in a 24‐year‐old male with newly suspected AML. We also present a systematic review of cases of HLH antecedent to, concurrent with, or sequel to AML to deduce trends in diagnosis, cytogenetic analysis, treatment, and outcome. This case highlights the importance of maintaining an increased index of suspicion for HLH among patients with malignancy and concomitant infectious exposures, particularly in dengue‐endemic regions.

This case report was prepared in accordance with the CARE (CAse REport) guidelines to ensure accurate, transparent, and complete reporting of clinical information [10].

## 2 Case Presentation

A previously healthy 24‐year‐old male patient presented to our institution with 2 weeks of high‐grade fever, tiredness, and anorexia, following a recent febrile illness clinically compatible with dengue infection, supported by dengue IgG seropositivity (IgM negative). The patient resided in a dengue‐endemic region, which increases the pretest probability of dengue infection in the setting of a compatible clinical presentation. He then progressed to develop pancytopenia with easy bruising.

On the first clinical assessment, the patient was observed to be pale and lethargic but was hemodynamically stable. No lymphadenopathy, hepatosplenomegaly, skin rashes, or neurological deficits were noted. Laboratory work‐ups revealed that the patient has severe pancytopenia with a WBC count of 2.52 × 10^9^/L, Hb of 5.46 g/dL, and platelet count of 7.41 × 10^9^/L. Severe elevation in the inflammatory markers was indicated with severely elevated serum ferritin levels of more than 2000 ug/L, with a peak at 33511 ug/L. The coagulation study revealed that the patient has severe coagulopathy with hypofibrinogenemia at 0.4 g/L, extremely high levels of serum D‐dimer at more than 40 mg/L FEU, elevated prothrombin time at 21.6 s, and INR at 1.62. Elevated liver enzymes were also present with AST and GGT at 294 and 118.6 IU/L, respectively. The patient was severely deficient in vitamin B12 at less than 148 pg/mL, with normal levels at 187–883 pg/mL. The complete laboratory workups can be seen in Table 1. These findings raised a strong clinical suspicion for HLH despite incomplete fulfillment of formal diagnostic criteria.

**TABLE 1:** Laboratory investigations on admission.

Parameter	Result	Reference range
WBC	2.52 × 10^9^/L	4–10
Hemoglobin	5.46 g/dL	13–17
Platelets	7.41 × 10^9^/L	140–440
Ferritin	> 2000 μg/L (peak 33 511)	4.6–204
Fibrinogen	Low	1.8–4.0 g/L
LDH	> 4500 U/L	125–220
D‐dimer	> 40 mg/L FEU	0–0.5
PT	21.6 s	11.7–15.3
INR	1.62	0.9–1.1
AST	294 U/L	5–34
ALT	24 U/L	0–55
GGT	118.6 U/L	12–64
Vitamin B12	< 148 pg/mL	187–883
Dengue serology	IgG+/IgM−	—
EBV, CMV	Negative	—

Owing to the chronic nature of the fever and the presence of neutropenia, the complete infection workup was conducted. Blood culture, urine culture, and septic screen were negative for bacteria. The serologies for Epstein–Barr virus infection and cytomegalovirus infection were negative. There was no evidence of sepsis either clinically or in the laboratory. The tuberculosis screen had been initiated but was pending at the time of the hospital transfer. The above findings made it less likely that the infection was the cause of the SIRS.

The peripheral smear was characterized by severe cytopenias, with blasts being scarce, but with a high monocytic differential and schistocytes. Bone marrow aspirate and biopsy were performed, searching for the presence of hemophagocytosis and to exclude malignancy. Bone marrow aspirate and biopsy revealed a markedly increased blast population (78%), with features suggestive of AML with monocytic differentiation based on flow cytometry. However, definitive classification according to WHO criteria could not be established, as complete cytogenetic, fluorescence in situ hybridization, and molecular studies were not available at the time of evaluation. Bone marrow photomicrography representative samples will not be included in this presentation, as the laboratory pictures from the archives are not available.

The patient was started on specific treatment for HLH with high doses of intravenous dexamethasone and intravenous immunoglobulin at a dose of 1 g/kg for two successive days. The empirical broad‐spectrum antibiotics were started for neutropenic sepsis. The patient also underwent aggressive supportive care for neutropenic sepsis with the administration of packed red cell units, platelets, and fresh frozen plasma in view of the coagulopathy.

During his hospitalization, he acquired disseminated intravascular coagulation, necessitating his transfer to an intensive care unit. However, his coagulation factors gradually returned to normal with transfusional therapy. With a suspected diagnosis of acute leukemia and HLH, and upon the request of his relatives, his urgent transfer to a tertiary care facility could be arranged. His subsequent management is not available to the authors.

## 3 Discussion

HLH is a devastating and life‐threatening hyperinflammatory syndrome that results from excessive and inappropriate activation of the immune system. It is characterized by chronic cytotoxic T lymphocyte and macrophage activation and proliferation that result in uncontrolled cytokine storm and, consequently, tissue damage. HLH is inherited (familial) or acquired (secondary), with the latter being infection, autoimmune disorders, malignancies, or pharmacologic drugs in origin [11]. While HLH has been clearly defined in children, its occurrence in adults and particularly in malignancies has been appreciated only in recent times. Hematologic malignancies are most commonly affected in M‐HLH, and among these, T‐cell and NK‐cell lymphomas are most commonly implicated. In comparison, AML‐associated HLH is rare, and only a few reports have been published describing its clinical presentation either before, coincident with, or after AML diagnosis [12].

The following case report indicates a diagnostically and therapeutically demanding case of HLH caused by dengue virus infection in a patient with suspected AML. This is a very rare association and, to the best of our knowledge, one of the rarest in the literature in which dengue fever had been a viral co‐inducer for HLH in an AML patient. The patient did not fulfill the complete HLH‐2004 diagnostic criteria, meeting four of the eight parameters (fever, cytopenias affecting greater than or equal to two lineages, hyperferritinemia, and hypofibrinogenemia). The remaining criteria, including splenomegaly, hypertriglyceridemia, soluble CD25 levels, NK cell activity, and indication of hemophagocytosis, were either absent or not assessed. However, in adult populations, strict adherence to HLH‐2004 criteria may delay diagnosis, and treatment is often initiated based on strong clinical suspicion. The HScore, a validated diagnostic tool for secondary HLH in adults, could have further supported the diagnosis; however, complete data required for its calculation were not available. Given the patient's rapidly progressive clinical course and characteristic laboratory abnormalities, a working diagnosis of HLH was made, and prompt treatment was initiated [13]. The concomitant vitamin B12 deficiency may have contributed to the severity of cytopenias and further complicated the clinical picture, although it is not a diagnostic marker for HLH. Although the patient did not fulfill all HLH‐2004 criteria, increasing evidence supports that adult HLH may present incompletely, and early treatment is often initiated based on strong clinical suspicion rather than strict fulfillment of all diagnostic parameters [3, 4]. In this particular case, though bone marrow aspiration and biopsy were done, none of the representative histopathological pictures were available from the laboratory archives, and hence, morphological documentation was impossible. However, it is important to note that the lack of histological confirmation does not negate the diagnosis of HLH because guidelines highlight that HLH is a clinical diagnosis made by using laboratory and clinical findings combined. In our patient, flow cytometry raised a strong suspicion for acute monocytic leukemia; however, definitive molecular confirmation was pending at the time of transfer to a tertiary care center. While the temporal relationship between the preceding illness and HLH is suggestive, a direct causal link cannot be definitively established based on the available data.

AML‐associated HLH is a unique subset of secondary HLH. Throughout our extensive review of the literature, we identified three broad temporal relationships of HLH and AML: HLH preceding AML, as shown in Table 2, HLH coinciding with AML, and HLH occurring during or after AML treatment. HLH‐AML has been reported predominantly in children after prior therapy with etoposide at high doses for infection‐associated HLH. Pan et al., for example, documented a 10‐month‐old girl who was treated with etoposide (cumulative 2100 mg/m^2^) for EBV‐associated HLH and subsequently developed AML with *t*(11;19)(q23;p13) at 23 months. In turn, Rama Chandran and Ariffin reported several cases of secondary AML, some presenting as acute promyelocytic leukemia (APL), after months to years from treatment with etoposide‐containing regimens for EBV‐HLH. Cytogenetic analysis in these cases most commonly demonstrates 11q23 translocations and chromosomal increases like +8 or +17, predisposing to an unbroken mutagenic pathway through topoisomerase II inhibition [14, 15, 16].

**TABLE 2:** Cases where HLH was diagnosed before the development of AML, commonly in children with EBV infections and often treated with etoposide.

Study	Age/sex	HLH trigger	Time to AML	AML type	Cytogenetics	AML treatment	Outcome
Pan et al. [14]	10 months/F	EBV	23 months later	AML (M4)	*t*(11;19)(q23;p13)	FLAG	Died during induction
Rama Chandran et al. [15]	5 years/F	EBV	9 months later	APL	*t*(15;17)(q22;q12)	ATRA + chemotherapy	Alive, in remission
Kitazawa et al. [16]	4 years/F	EBV	31 months	FAB M2 AML	Normal karyotype	Allogeneic HSCT	Died posttransplant

*Note:* MLL‐rearrangements (11q23) were frequent. AML onset occurred within 1–2 years of HLH diagnosis.

HLH contemporaneously diagnosed with suspected AML, as in our case, is even more rarely documented and almost always has a dire prognosis, as shown in Table 3. In one such case reported by Takahashi et al., a 19‐year‐old female patient of virus‐associated HLH was also found to have AML with *t*(9;11)(p22;q23) translocation occurring simultaneously and died after HSCT. Vergara‐Lluri and Chan's report was a case of a 15‐year‐old patient with acute megakaryoblastic leukemia (AML‐M7) who presented as HLH, who did not respond to conventional HLH or AML treatment, and who died due to progressive disease. The cytogenetic findings in our patient (*t*(1;9)(q42;q22), add(11)(q23)) are suggestive of clonal hematologic disease; however, in the absence of complete molecular characterization, their definitive diagnostic and prognostic significance remains uncertain. From our experience, it is evident that cytogenetic abnormalities in concomitant HLH–AML cases are heterogeneous but feature high‐risk features such as complex karyotypes, trisomies, or MLL‐involving chromosomal translocations [17, 18, 19].

**TABLE 3:** Cases where HLH and AML were diagnosed simultaneously.

Study	Age/sex	HLH trigger	AML subtype	Cytogenetics	HLH therapy	AML therapy	Outcome
Takahashi et al. [17]	19 years/F	Virus‐associated	AML	*t*(9;11)(p22;q23)	HLH‐94	HSCT	Died
Vergara‐Lluri and Chan [18]	15 years/M	Not specified	AML‐M7	Complex karyotype	HLH‐94	AML chemo	Died
Hatano et al. [19]	36 years/F	Not stated	Monocytic AML	Not specified	Supportive	AML chemo	Alive
Present case	24 years/M	Dengue	AML (M4)	*t*(1;9)(q42;q22), add(11)(q23)	IVIG, supportive	Not given	Unknown (patient transferred to tertiary care center; follow‐up unavailable)

*Note:* Most presented with severe inflammation and coagulopathy. Cytogenetic abnormalities were often present, especially involving 11q23. Outcomes were generally poor.

HLH following AML treatment, specifically in relapse or induction, is increasingly reported, most significantly in children with myelomonocytic or monocytic AML subtypes, as shown in Table 4. Lackner et al. described a series of children with HLH developing during or following chemotherapy for AML with translocation (9;11) that was responsive to dexamethasone and cyclosporine but required withdrawal of antileukemic therapy. There are also reports of HLH secondary to infection (e.g., CMV, Candida) or HLH secondary to bone marrow regeneration, usually in the context of chemotherapy‐induced myelosuppression. Adults have been reported by Chen et al. with fulminant HLH in an adult AML‐M5 patient after IA induction therapy, with multisystem failure and death despite treatment with high‐dose steroids and IVIG. These posttreatment HLH cases highlight the delicate balance between oncologic control and hyperinflammation control, particularly in patients with active cytopenias and impaired immune surveillance [20, 21, 22, 23].

**TABLE 4:** Cases where HLH occurred after AML diagnosis or during chemotherapy.

Study	Age/sex	AML subtype	HLH trigger	Cytogenetics	AML therapy	HLH treatment	Outcome
Lackner et al. [20]	Pediatric cohort	AML with *t*(9;11)	Post‐chemo	*t*(9;11)	Standard AML protocol	Dexamethasone, cyclosporine	Survived
Zhang et al. [21]	Mixed adult; 3 patients (58, etc.)	AML transformed from MDS, AML‐M5/M2	During induction chemo	Various	Decitabine + CAG, and so forth	Dexamethasone, cyclosporine, etoposide	Two died (19 and 27 days), one survived 4 months
Zorzetto et al. [22]	Adult	Relapsed/refractory AML	Persistent HLH while relapsed	Not specified	Azacitidine + venetoclax	Azacitidine + venetoclax	Resolved HLH
Delavigne et al. [23]	Adult	AML under intensive chemo	Infection‐related, neutropenia	Various	Standard induction	Supportive ± steroids	Poor prognosis highlighted

*Note:* Triggers included infections and marrow regeneration. Prognosis was variable, depending on infection control and immune response.

The possibility of dengue infection in our patient represents an important but not definitively established contributing factor. Although dengue IgG seropositivity alone does not confirm acute infection, the patient had a recent febrile illness clinically compatible with dengue and resided in a dengue‐endemic region, which increases the likelihood of dengue exposure. The temporal relationship between this illness and the onset of hyperinflammatory features suggests a possible role of a viral trigger in precipitating HLH. However, in the absence of confirmatory testing such as NS1 antigen, RT‐PCR, or IgM serology, recent dengue infection cannot be definitively established. Therefore, it is more appropriate to interpret this case as HLH occurring in the context of a recent viral illness compatible with dengue rather than conclusively triggered by dengue infection. Dengue infection in this context could have the secondary role of enhancing the pro‐inflammatory potential of AML, especially in patients with monocytic differentiation, who are immunoreactive [24]. This temporal association between recent dengue infection and the onset of hyperinflammatory features strongly supports the role of dengue as a triggering event for secondary HLH in this patient. Although dengue IgG positivity alone cannot confirm recent infection, the patient's epidemiologic exposure and temporal relationship between febrile illness and onset of hyperinflammatory features suggest that dengue may have acted as a possible co‐trigger for HLH.

Treatment management for M‐HLH is clinically debated. While HLH‐94 treatment with dexamethasone and etoposide is the standard treatment in primary and viral HLH, it is used in M‐HLH very infrequently due to the fear of additional marrow suppression and disruption of oncologic therapy. Modern practice now endorses treatment of the underlying tumor with or without immunomodulatory therapy, that is, corticosteroids, IVIG, and plasma exchange, with HLH‐targeted agents in refractory disease. Our patient was not eligible for cytotoxic chemotherapy at presentation due to coagulopathy and critical illness. He was treated with IVIG, fresh frozen plasma, and supportive transfusions but kept worsening, indicating the fulminant course of M‐HLH and early diagnosis and treatment [25]. In our patient, early initiation of dexamethasone and intravenous immunoglobulin, together with aggressive correction of coagulopathy, resulted in initial clinical stabilization before transfer. Despite the established role of etoposide‐containing regimens for the management of both primary and infection‐associated HLH, their use within M‐HLH would require careful evaluation. The decision not to employ etoposide infusions in our case would be due to the presence of severe cytopenias, active coagulopathy, and hemodynamic instability, thereby increasing the risk for therapy‐related complications. The approach would, however, involve supportive care for hemodynamic stability before the initiation of definitive cancer therapy protocols.

The case indicates the necessity of a high suspicion of HLH in the context of patients with persistent fever, cytopenias, liver impairment, and highly elevated hyperferritinemia, particularly in the context of hematologic malignancy and recent viral infection. It emphasizes the need for expedient diagnostic workup and multimodal treatment strategies to balance oncologic control and immunosuppression. It adds to the meager evidence base for HLH caused by dengue in adult patients and extends the phenotypic range of HLH in AML. Future prospective studies are needed to determine the optimal diagnostic criteria, prognostic factors, and treatment regimens for HLH in adult AML patients [26].

This case has several limitations. First, histopathological documentation of bone marrow findings could not be included due to the unavailability of archived micrographs. Second, although flow cytometry suggested acute monocytic leukemia, complete molecular and cytogenetic characterization was not available at the time of transfer. Also, long‐term clinical outcome could not be assessed, as follow‐up data after referral to a tertiary center were not accessible. In addition, the absence of confirmatory virological testing (such as NS1 antigen, RT‐PCR, or IgM serology) limits the ability to establish dengue as a definitive trigger in this case. Furthermore, the lack of complete cytogenetic and molecular characterization limited definitive classification of AML according to WHO criteria.

## 4 Conclusion

This case highlights the importance of considering HLH in patients with unexplained systemic inflammation, particularly when there is a known hematologic malignancy and recent viral illness. Early recognition and intervention are critical, though prognosis remains guarded in patients with concurrent HLH and AML.

## Author Contributions

**Sariya Khan:** writing – original draft, writing – review and editing. **Halima Saleh:** conceptualization, visualization, writing – review and editing. **Sahar Albraim:** investigation, writing – review and editing, writing – original draft. **Amatul Rafey:** writing – original draft. **Huda Albraim:** conceptualization, writing – original draft. **Mable Pereira:** supervision, project administration, writing – review and editing, writing – original draft. **Tarek Mohammed:** conceptualization, writing – review and editing, supervision, project administration.

## Funding

The authors have nothing to report.

## Ethics Statement

Ethical approval has been exempted by our institution since this is a case report, and no new studies or new interventions were carried out.

## Consent

The authors have nothing to report.

## Conflicts of Interest

The authors declare no conflicts of interest.

## Data Availability

The data that support the findings of this study are available on request from the corresponding author. The data are not publicly available due to privacy or ethical restrictions.

## References

[1] H. Al‐Samkari and N. Berliner , “Hemophagocytic Lymphohistiocytosis,” Annual Review of Pathology: Mechanisms of Disease 13 (2018): 27–49. 10.1146/annurev-pathol-020117-043625 28934563

[2] G. Griffin , S. Shenoi , and G. C. Hughes , “Hemophagocytic Lymphohistiocytosis: An Update on Pathogenesis, Diagnosis, and Therapy,” Best Practice & Research. Clinical Rheumatology 34, no. 4 (2020): 101515. 32387063 10.1016/j.berh.2020.101515

[3] J. I. Henter , A. Horne , M. Aricó , et al., “HLH‐2004: Diagnostic and Therapeutic Guidelines for Hemophagocytic Lymphohistiocytosis,” Pediatric Blood & Cancer 48, no. 2 (2007): 124–131, 10.1002/pbc.21039. 16937360

[4] A. M. Schram and N. Berliner , “How I Treat Hemophagocytic Lymphohistiocytosis in the Adult Patient,” Blood 125, no. 19 (2015): 2908–2914, 10.1182/blood-2015-01-551622. 25758828

[5] A. H. Filipovich , “Hemophagocytic Lymphohistiocytosis (HLH) and Related Disorders,” ASH Education Program 2009, no. 1 (2009): 127–131. 10.1182/asheducation-2009.1.127 20008190

[6] M. Ramos‐Casals , P. Brito‐Zerón , A. López‐Guillermo , M. A. Khamashta , and X. Bosch , “Adult Haemophagocytic Syndrome,” Lancet 383, no. 9927 (2014): 1503–1516, 10.1016/S0140-6736(13)61048-X. 24290661

[7] K. Risma and M. B. Jordan , “Hemophagocytic Lymphohistiocytosis: Updates and Evolving Concepts,” Current Opinion in Pediatrics 24, no. 1 (2012): 9–15, 10.1097/MOP.0b013e32834ec9c1. 22189397

[8] P. La Rosée , A. Horne , M. Hines , et al., “Recommendations for the Management of Hemophagocytic Lymphohistiocytosis in Adults,” Blood 133, no. 23 (2019): 2465–2477. 30992265 10.1182/blood.2018894618

[9] M. Aricò , C. Danesino , D. Pende , and L. Moretta , “Pathogenesis of Haemophagocytic Lymphohistiocytosis,” British Journal of Haematology 114, no. 4 (2001): 761–769. 11564062 10.1046/j.1365-2141.2001.02936.x

[10] D. S. Riley , M. S. Barber , G. S. Kienle , et al., “CARE Guidelines for Case Reports: Explanation and Elaboration Document,” Journal of Clinical Epidemiology 89 (2017): 218–235, 10.1016/j.jclinepi.2017.04.026. 28529185

[11] J. C. Lee and A. C. Logan , “Diagnosis and Management of Adult Malignancy‐Associated Hemophagocytic Lymphohistiocytosis,” Cancers 15, no. 6 (2023): 1839. 36980725 10.3390/cancers15061839 PMC10046521

[12] N. Daver , K. McClain , C. E. Allen , et al., “A Consensus Review on Malignancy‐Associated Hemophagocytic Lymphohistiocytosis in Adults,” Cancer 123, no. 17 (2017): 3229–3240. 28621800 10.1002/cncr.30826 PMC5568927

[13] G. Insuasti‐Beltran and N. S. Rosenthal , “Bone Marrow Findings in Inflammatory, Infectious, and Metabolic Disorders,” in Hematopathology, ed. A. Orazi , D. A. Arber , E. S. Jaffe , E. Campo , L. M. Rimsza , and S. H. Swerdlow (Elsevier, 2024), 250–268.

[14] H. Pan , D. N. Feng , L. Song , and L. R. Sun , “Acute Myeloid Leukemia Following Etoposide Therapy for EBV‐Associated Hemophagocytic Lymphohistiocytosis: A Case Report and a Brief Review of the Literature,” BMC Pediatrics 16, no. 1 (2016): 116. 27473573 10.1186/s12887-016-0649-z PMC4967337

[15] S. RamaChandran and H. Ariffin , “Secondary Acute Myeloid Leukemia After Etoposide Therapy for Haemophagocytic Lymphohistiocytosis,” Pediatric Blood & Cancer 53, no. 3 (2009): 488–490. 19434733 10.1002/pbc.22063

[16] J. Kitazawa , E. Ito , K. Arai , M. Yokoyama , M. Fukayama , and S. Imashuku , “Secondary Acute Myelocytic Leukemia After Successful Chemotherapy With Etoposide for Epstein–Barr Virus‐Associated Hemophagocytic Lymphohistiocytosis,” Medical and Pediatric Oncology 37, no. 2 (2001): 153–154. 11496359 10.1002/mpo.1189

[17] T. Takahashi , F. Yagasaki , K. Endo , et al., “Therapy‐Related AML After Successful Chemotherapy With Low Dose Etoposide for Virus‐Associated Hemophagocytic Syndrome,” International Journal of Hematology 68, no. 3 (1998): 333–336. 9846019 10.1016/s0925-5710(98)00070-x

[18] M. Vergara‐Lluri and R. Y. Chan , “Acute Megakaryoblastic Leukemia Presenting as Hemophagocytic Lymphohistiocytosis,” Journal of Pediatric Hematology/Oncology 42, no. 1 (2020): 61–62. 31714436 10.1097/MPH.0000000000001660

[19] K. Hatano , T. Nagai , T. Matsuyama , et al., “Leukemia Cells Directly Phagocytose Blood Cells in AML‐Associated Hemophagocytic Lymphohistiocytosis: A Case Report and Review of the Literature,” Acta Haematologica 133, no. 1 (2014): 98–100. 25226795 10.1159/000360840

[20] H. Lackner , M. G. Seidel , V. Strenger , et al., “Hemophagocytic Syndrome in Children With Acute Monoblastic Leukemia—Another Cause of Fever of Unknown Origin,” Supportive Care in Cancer 21, no. 12 (2013): 3519–3523. 23975227 10.1007/s00520-013-1937-x

[21] H. Zhang , Y. Wang , S. Feng , et al., “Case Report Hemophagocytic Lymphohistiocytosis in Patients With Acute Myeloid Leukemia: A Report of Three Cases and Literature Review,” International Journal of Clinical and Experimental Medicine 11, no. 8 (2018): 8788–8792.

[22] F. Zorzetto , A. Scalas , F. Longu , M. A. Isoni , E. Angelucci , and C. Fozza , “Hemophagocytic Lymphohistiocytosis Secondary to Refractory Acute Myeloid Leukemia Resolved After Second‐Line Treatment With Azacitidine Plus Venetoclax,” Mediterranean Journal of Hematology and Infectious Diseases 16, no. 1 (2024): e2024011. 38223479 10.4084/MJHID.2024.011 PMC10786137

[23] K. Delavigne , E. Bérard , S. Bertoli , et al., “Hemophagocytic Syndrome in Patients With Acute Myeloid Leukemia Undergoing Intensive Chemotherapy,” Haematologica 99, no. 3 (2014): 474. 24142998 10.3324/haematol.2013.097394 PMC3943310

[24] D. Zhao , L. Qian , and J. Shen , “Acute Myelocytic Leukemia in a Patient With Hemophagocytic Lymphohistiocytosis: A Case Report,” Oncology Letters 8, no. 6 (2014): 2634–2636. 25364441 10.3892/ol.2014.2527 PMC4214506

[25] M. Machaczka , H. Nahi , H. Karbach , M. Klimkowska , and H. Hägglund , “Successful Treatment of Recurrent Malignancy‐Associated Hemophagocytic Lymphohistiocytosis With a Modified HLH‐94 Immunochemotherapy and Allogeneic Stem Cell Transplantation,” Medical Oncology 29, no. 2 (2012): 1231–1236. 21533602 10.1007/s12032-011-9963-3

[26] Z. K. Otrock and C. S. Eby , “Clinical Characteristics, Prognostic Factors, and Outcomes of Adult Patients With Hemophagocytic Lymphohistiocytosis,” American Journal of Hematology 90, no. 3 (2015): 220–224. 25469675 10.1002/ajh.23911

